# Treatment burden in glomerular diseases: advances and challenges in immunosuppressive therapy

**DOI:** 10.3389/fneph.2025.1545373

**Published:** 2025-05-01

**Authors:** Mythri Shankar, Tanuj Moses Lamech

**Affiliations:** ^1^ Department of Nephrology, Institute of Nephrourology, Bengaluru, Karnataka, India; ^2^ Department of Nephrology, SRM Medical College Hospital and Research Centre, Kattankulathur, Tamilnadu, India

**Keywords:** glomerular diseases, kidney diseases, infection, adverse effects, treatment burden, economic burden

## Abstract

Glomerular diseases represent a significant global health challenge, complicated by the intricate management required for their treatment. We examine the treatment burden associated with the immunosuppressive therapies used to manage these conditions, focusing on the efficacy, side effects, and financial implications of commonly used medications such as glucocorticoids, mycophenolate mofetil (MMF), cyclophosphamide, calcineurin inhibitors and Rituximab. Immunosuppressive treatments, while effective in controlling disease activity, can result in a variety of adverse effects ranging from gastrointestinal symptoms and bone marrow suppression to increased infection risks, necessitating careful monitoring and dose adjustments to mitigate these risks. Hence, the need for a balanced approach in therapy management, incorporating regular monitoring and potential dose modifications to enhance patient outcomes while minimizing side effects. Additionally, these treatments have an economic impact, particularly in lower-income regions where access to medication and the cost of medication can limit patient outcomes. There have been certain advancements in treatment modalities, such as the use of enteric-coated formulations and tailored dosing schedules, which aim to improve drug tolerability and adherence. By addressing these critical aspects, we aim to shed light on the ongoing challenges and developments in the management of glomerular diseases, emphasizing the need for continued research and innovation in therapeutic strategies to reduce the overall treatment burden and improve the quality of life for affected individuals.

## Introduction

Chronic kidney disease (CKD) represents a major global public health challenge due to its role in increasing the risk of kidney failure and various related complications. As of 2019, CKD ranked 18th in terms of global disability-adjusted life-years (DALYs) lost, accounting for approximately 2% of the worldwide DALYs. Between 1990 and 2019, the number of DALYs attributed to CKD surged by 93%, from 21.5 million to 41.5 million. Projections suggest that CKD will become the 13th leading cause of death by 2030 and rise to the fourth most common cause of death by 2040. The 2019 Global Burden of Disease (GBD) Study categorized CKD into five types based on its underlying causes: type 1 and type 2 diabetes, hypertension, glomerulonephritis, and other unspecified reasons.

The epidemiology of CKD due to glomerulonephritis differs depending on geographic and demographic factors. While diabetes and hypertension are the primary causes of CKD in developed nations, glomerulonephritis remains the most prevalent cause in many lower-income regions, particularly in parts of Asia and sub-Saharan Africa.

In 2019, there were an estimated 606,300 new cases of CKD due to glomerulonephritis worldwide, with a prevalence of 17.3 million cases. The disease caused around 183,700 deaths and led to 6.9 million DALYs. These figures represent substantial increases since 1990, with incident cases rising by 77%, prevalent cases by 81%, deaths by 100%, and DALYs by 66%. The burden of disease was particularly concentrated in regions with lower sociodemographic indices. Central Latin America showed a disproportionately higher disease burden relative to its sociodemographic index. Analysis indicates that population growth and aging were the primary contributors to the increase in DALYs. Notably, while most of the burden was among middle-aged and elderly individuals, the highest incidence was observed in children aged 1–4 years, revealing an opportunity for intervention to reduce age-standardized DALYs in middle-sociodemographic regions (1).

Infections significantly contribute to morbidity and mortality in patients with glomerular disease. Adults and children with confirmed glomerular disease were monitored over time as part of the Cure Glomerulonephropathy Network. The findings indicated that exposure to various immunosuppressive drugs, particularly those including corticosteroids, was linked to a higher risk of infection, while exposure to purine inhibitors alone did not seem to significantly elevate that risk (2).

## Glucocorticoids

They are a broad class of medications widely used in medicine for their potent anti-inflammatory and immunosuppressive properties. Their mechanism of action is complex and involves several pathways at both the cellular and molecular levels:

1. Modulation of gene expression: Corticosteroids enter cells and bind to the glucocorticoid receptor in the cytoplasm. This complex then translocates into the nucleus, where it can bind to glucocorticoid response elements (GREs) in the DNA. This binding can either upregulate or downregulate the transcription of specific genes that are involved in inflammatory processes.
2. Inhibition of transcription factors: Corticosteroids can inhibit the activity of transcription factors such as nuclear factor-kappa B (NF-κB) and activator protein 1 (AP-1), both of which play critical roles in the inflammatory response.
3. Suppression of the immune response: Steroids affect various types of white blood cells and their function. They can reduce the proliferation of T cells and cause apoptosis (programmed cell death) of certain immune cells, thereby dampening the immune response. They also inhibit the function of antigen-presenting cells and decrease the secretion of cytokines by immune cells, further reducing inflammation.
4. Stabilization of cellular membranes: Corticosteroids stabilize lysosomal membranes, which can prevent the release of proteolytic enzymes that contribute to tissue inflammation and damage when cells are destroyed.
5. Inhibition of arachidonic acid metabolism: Corticosteroids inhibit phospholipase A2, an enzyme critical for the release of arachidonic acid from cell membrane phospholipids. Arachidonic acid is a precursor for the synthesis of eicosanoids, including prostaglandins and leukotrienes, which are potent mediators of inflammation. By inhibiting this pathway, steroids reduce the synthesis of these inflammatory mediators.

GCs possess numerous side effects, such as an increased risk of infection, bone disorders, dysglycemia, obesity, hypertension, psychosis, gastrointestinal bleeding, cataracts, and long-term cardiovascular disease risks.

### Infections

Specific infections that should be considered for screening and prophylaxis include Pneumocystis jirovecii pneumonia (PJP), tuberculosis, strongyloidiasis, hepatitis B (HBV) and C (HCV), HIV, herpes zoster virus, and candidiasis. Observational studies involving patients with rheumatic diseases have shown that low-dose glucocorticoids (such as prednisone below 10 mg per day) carry a slightly elevated risk of bacterial infections. However, the risk of more severe opportunistic infections increases with higher doses (prednisone over 20 mg per day). In patients with glomerular diseases, the risk of infection may be further heightened due to urinary loss of immunoglobulins and complement, as well as the immunocompromised state associated with kidney disease (3).

### Osteonecrosis

Glucocorticoids (GCs) lead to accelerated bone loss initially during the first 3–6 months, which continues as a sustained reduction in bone formation throughout the treatment period. The risk of fractures is influenced by both the highest and total cumulative dose of GCs, and remains elevated even with chronic low doses, such as 5 mg of prednisone daily. For patients on long-term steroids, the 2017 guidelines from the American College of Rheumatology advise assessing fracture risk through bone mineral density tests and the use of an online tool called FRAX (available at https://www.shef.ac.uk/FRAX/tool.jsp) for adults over the age of 40. It is recommended that all patients receive oral calcium (1000–1200 mg/day) and vitamin D (600–800 IU/day). Oral bisphosphonates are advised for individuals with a moderate to high risk of fractures (10-year fracture risk of 10%–20%) (4).

### Avascular necrosis

Steroid therapy can lead to a serious condition known as avascular necrosis (osteonecrosis), often seen in the femoral head. This condition is generally linked to ischemia resulting from lipid metabolism irregularities, oxidative stress, and vascular damage. It is particularly common among younger patients with Systemic Lupus Erythematosus (SLE), likely due to their chronic inflammatory and procoagulant states. Symptoms such as hip pain typically manifest 2-3 years after beginning GC treatment, though they can appear sooner. Unlike GC-induced osteoporosis, avascular necrosis is uncommon in patients who receive peak daily doses of prednisone below 20-30 mg.

### Adrenal suppression

Long-term use of GCs can inhibit the hypothalamic-pituitary-adrenal (HPA) axis through a negative feedback mechanism on corticotropin-releasing hormone and corticotropin. This suppression can persist even after reducing or discontinuing GC therapy, leading to adrenal insufficiency. Symptoms like nausea and fatigue are common with low cortisol levels; however, in acute stress situations such as surgery, an adrenal crisis could occur, prompting the need for stress-dose steroids perioperatively in high-risk patients. Observational studies report a wide variation in the prevalence of biochemical adrenal insufficiency (14%-63%), though clinical adrenal insufficiency is rare (5, 6). The risk of adrenal insufficiency is minimal if the duration of GC use is less than three weeks. To prevent adrenal insufficiency, GCs are typically administered in the morning and may be prescribed on alternate days. With prolonged use, the dose can be tapered relatively quickly to a physiological level (around 7.5 mg/day), but tapering should be slower once below this level.

## Mycophenolate mofetil

MMF and enteric-coated mycophenolate sodium function as antiproliferative agents by targeting inosine monophosphate dehydrogenase, which is critical in the purine synthesis pathway of both B and T lymphocytes. These drugs are largely protein-bound (97%) and conditions like hypoalbuminemia associated with nephrotic syndrome can elevate levels of free mycophenolic acid (MPA), potentially heightening the risk of adverse effects. Importantly, the absorption of these medications can be significantly reduced by antacids or phosphate binders (up to 33%) and proton pump inhibitors (by 25% or more), which may lead to decreased effectiveness and fewer complications. KDIGO recommends MMF for

- The management of C3 glomerulopathy after ruling out monoclonal gammopathy, along with steroids, in patients with proteinuria >1gram/day and hematuria or a declining kidney function for a period of at least 6 months.
- Maintenance therapy for Lupus nephritis (MMF - 2g/day for not less than 36 months including induction phase) (**Table 1**).
- Maintenance therapy for ANCA vasculitis (MMF - 2g/day at complete remission for 2 years).
- Steroid dependent nephrotic syndrome or frequently relapsing nephrotic syndrome as steroid sparing agent.
- In IgA nephropathy as steroid-sparing agent in Chinese population (6).

**Table 1:** Indications for Mycophenolic Acid Analogs (MPAA) in Lupus Nephritis (KDIGO 2024) (7).

Clinical Setting	Indication	Remarks
Initial Therapy	For active Class III or IV LN (± membranous component) and for active class V LN	- Can be used with glucocorticoids- Strong preference due to efficacy and safety profile
	Choice between MPAA, low-dose intravenous cyclophosphamide, or belimumab	- Based on individual patient factors, including side effect profiles and patient preference
Maintenance Therapy	After completion of initial therapy, continued use of MPAA for maintenance	- Recommended to reduce risk of flare-ups and maintain remission
	Alternative to cyclophosphamide or azathioprine for maintenance	- Preferred due to better tolerability and efficacy in maintaining remission
Special Considerations	Preferred initial therapy for patients at high risk of infertility	- Lower gonadotoxicity compared to cyclophosphamide, important for younger patients or those considering future fertility

### Infections

Treatment with MMF particularly increases the risk of viral infections such as herpes zoster, cytomegalovirus, and herpes simplex, which is exacerbated by bone marrow suppression and leukopenia. In contrast, infections from hepatitis B (HBV) and hepatitis C (HCV) viruses may not significantly increase, as mycophenolic acid (MPA) can inhibit the expression of the HBV surface antigen and reduce HBV viral replication (8). Additionally, MPA might suppress P. jirovecii infections. In a review of four studies involving kidney transplant recipients, none of the 1068 patients treated with MMF developed Pneumocystis jirovecii pneumonia (PJP), whereas ten out of 563 patients on other types of immunosuppression, primarily azathioprine, did contract PJP (9).

Gastrointestinal (GI) symptoms and dose-dependent bone marrow suppression are the other most common side effects observed, yet these often improve with adjustments in dosage. In comparison to cyclophosphamide, MMF is generally safer under most conditions. Unlike with azathioprine, thiopurine methyltransferase deficiency does not pose a concern when prescribing MMF. The most frequent adverse reaction to MMF is persistent diarrhea. Over time, these symptoms usually diminish and seldom led to the discontinuation of MMF. Years into the treatment, the incidence of GI symptoms decreases, with the remaining symptoms being minor. GI side effects occurred in patients treated for SLE, leading to drug discontinuation in up to 30 percent of cases (10). Some patients may find relief by taking smaller doses more frequently throughout the day, while others might need a lower total daily dose. For some, switching to enteric-coated mycophenolate sodium (EC-MPS) could be beneficial (11).

Histopathological alterations linked to GI symptoms in patients with glomerular diseases are not well defined but have been explored in a retrospective analysis of solid organ transplant recipients who underwent diagnostic colonic biopsies (12). In this group, 69 percent of the 32 patients on MMF (dosed at 500 to 1000 mg twice daily) displayed abnormal findings, including indicators of inflammatory bowel disease (28 percent), graft-versus-host disease (19 percent), ischemia (3 percent), or transient colitis (16 percent). In contrast, only one out of eight patients not taking MMF showed significant histopathological changes, presenting a mild pattern resembling graft-versus-host disease.

Bone marrow suppression is another significant risk, necessitating regular monitoring. It is recommended to perform complete blood counts (CBCs) after the initial one to two weeks of MMF treatment followed by checks every six to eight weeks thereafter, provided no cytopenias are detected.

The other side effects include headaches (9.3%), fatigue (5.7%), eczema (5%), and hair loss (3.5%).

## Azathioprine

Azathioprine is a purine analogue that inhibits purine nucleotide synthesis and interferes with the synthesis and metabolism of RNA. It continues to play an important role in certain subsets of patients with systemic lupus erythematosus such as pregnant women, and as a maintenance agent for ANCA-associated vasculitis (**Table 2**).

**Table 2:** Indications for Azathioprine in Lupus Nephritis (KDIGO 2024) (7).

Clinical Setting	Indication	Remarks
Maintenance Therapy	After induction therapy	Azathioprine is recommended as a maintenance treatment following induction therapy with MPAA or cyclophosphamide.
	Alternative to MPAA for maintenance	Suitable for patients who do not tolerate MPAA or when MPAA is contraindicated.
During Pregnancy	Safe alternative during pregnancy	Considered safer than other immunosuppressants for use during pregnancy.
Long-term Use	Minimize toxicity while maintaining remission	Used due to its relatively favorable safety profile for long-term treatment.

The clinically relevant adverse effect of azathioprine is bone marrow suppression, manifesting as leukopenia and/or thrombocytopenia, which warrants close monitoring during the initial weeks after initiation of therapy or escalation of dosage. Drug-induced cytopenias usually rapidly resolves on dose reduction or withdrawal of azathioprine. Co-prescription of azathioprine along with allopurinol should be avoided, or used only with great caution, as xanthine oxidase is the major pathway by which azathioprine is converted into inactive metabolites. Inhibition of this enzyme with allopurinol therefore puts the patient at a much higher risk of bone marrow toxicity (13).

Patients who have genetic polymorphisms that result in reduced thiopurine methyltransferase (TPMT) activity are also at higher risk for cytopenias, as this enzyme provides an alternative metabolic pathway by which azathioprine is converted into inactive metabolites. It is possible to both measure enzyme activity and identify genetic polymorphisms in TPMT, and this predicts bone marrow suppression with azathioprine. However, cytopenias can still occur even in patients with normal TPMT activity. Furthermore, the cost-effectiveness of TMPT screening for all patients before drug initiation, as compared with just routine monitoring of blood counts, remains unclear (14).

Other toxicities of azathioprine include hepatitis, cholestasis, and rarely pancreatitis.

## Cyclophosphamide

Cyclophosphamide, an old chemotherapeutic and immunosuppressive agent, remains relevant due to its cost-effectiveness and efficacy in particular clinical scenarios.

Cyclophosphamide is a prodrug activated in the liver, producing metabolites that cause DNA cross-linking and breaks, particularly effective in proliferating cells like lymphocytes. Its action can lead to cell apoptosis, important in controlling autoimmune activity in glomerular diseases.

Despite its side effects, cyclophosphamide is used in minimal change disease and focal segmental glomerulosclerosis as a steroid-sparing agent, and in membranous nephropathy and rapidly progressive glomerulonephritis as part of induction therapy. It’s particularly crucial in severe lupus nephritis, offering a cheaper alternative to newer agents (**Table 3**).

**Table 3:** Indications for Cyclophosphamide in Lupus Nephritis (KDIGO 2024) (7).

Clinical Setting	Indication	Remarks
Initial Therapy	For active Class III or IV LN	Cyclophosphamide is used in combination with glucocorticoids for severe cases of lupus nephritis.
	Alternative to MPAA	Chosen for patients who might not tolerate or respond to MPAA.
Maintenance Therapy	After induction therapy in severe cases	Used to maintain remission following initial control of the disease.
	High-risk patients	Recommended for patients with high risk of relapse or severe renal involvement.
Refractory Disease	For patients not responding to standard therapies	Considered in cases where there is an inadequate response to other immunosuppressive agents.

### Adverse effects

The majority of side effects associated with cyclophosphamide are related to the dosage and the age of the patient. Phosphoramide mustard, one of its metabolites, can lead to bone marrow and gonadal damage and may increase the risk of leukemia, bladder cancer, and other cancers. Another metabolite, acrolein, can cause severe bladder inflammation and scarring if used over extended periods. To minimize these harmful effects, several preventive actions are recommended. Notably, it is advised to limit the use of cyclophosphamide in long-term treatments and high dosages wherever feasible (15). It has been reported that patients who received more than 36 grams of cyclophosphamide have a 3.6 times higher risk of developing bladder cancer. In a retrospective analysis involving 1,018 patients treated with cyclophosphamide, fewer than 2% experienced hemorrhagic cystitis after a median duration of 10 months, while about 0.19% were diagnosed with bladder cancer after a median period of 8 years, across a total of 4,224 patient-years (16).

Cyclophosphamide can cause the germinal epithelium in the testes of males to stop functioning, potentially leading to reduced sperm count or complete lack of sperm. This side effect depends on the dosage, duration of treatment, and the patient’s age. A meta-analysis involving children with idiopathic nephrotic syndrome found a low risk of producing no sperm when the total dose remained below 250 mg/kg (17). For adults, it is advised to keep the cumulative dose under 168 mg/kg (18).

In females, the drug can lead to the cessation of menstruation and ovarian failure, with the length of treatment and total dosage being critical factors. In women with lupus nephritis, these are significant risk factors for menstruation stopping before menopause. Research on women treated with cyclophosphamide for breast cancer showed that the typical dose before menstruation stopped was 5.2 grams for women in their 40s and 9.3 grams for those in their 30s (13, 19).

### Precautionary measures

Management strategies to mitigate risks include using the lowest effective dose, frequent monitoring of blood counts, ensuring adequate hydration, and using protective agents like MESNA to prevent bladder toxicity. In certain cases, alternative agents are preferred to avoid the cumulative toxicities associated with cyclophosphamide.

While newer agents provide effective alternatives with potentially fewer side effects, cyclophosphamide’s efficacy in specific settings justifies its ongoing use. Its role in treatment protocols continues due to its ability to induce remission in severe cases and its cost-effectiveness, making it a valuable option in resource-constrained settings. However, careful patient selection and monitoring are imperative to minimize its risks.

## Calcineurin inhibitors

The introduction of cyclosporine in the 1980’s and tacrolimus in the 1990’s revolutionized the field of transplantation, and their use was subsequently extended to various glomerulonephritis (GN’s), including membranous nephropathy, lupus nephritis, and steroid-dependent and steroid-resistant nephrotic syndromes. However, despite their clinical efficacy, both drugs are associated with significant toxicities that often limit their use.

### Adverse effects

Of greatest concern to the nephrologist is the consistent association with nephrotoxicity. CNI’s constrict the afferent arteriole in a dose-dependent manner, resulting in a reversible decrease in GFR. However, given that target trough levels of CNI’s for GN’s are lower than in the transplant setting, this is likely a minor effect. More importantly, long-term use of CNI’s are associated with increasing interstitial fibrosis and tubular atrophy, and a consequent irreversible decline in GFR. This often limits the use of these drugs to a few years, necessitating a reliance on safer alternatives in the long term. Additionally, there are tubulotoxic effects that result in type 4 renal tubular acidosis (hyperkalemia, normal anion-gap metabolic acidosis), and hypomagnesemia (13).

Tacrolimus has specifically been associated with glucose intolerance and a worsening of glycemic control, compared to cyclosporine (20, 21). Similarly, the risk of neurotoxicity is also much higher with tacrolimus, manifesting most commonly as a coarse tremor, but sometimes progressing to seizures and posterior reversible encephalopathy syndrome (PRES). Cyclosporine, on the other hand, uniquely produces hypertrichosis and gingival hyperplasia, which is particularly problematic when used in young women. Hyperlipidemia and hypertension are more common with cyclosporine than tacrolimus. CNI's also increase the risk of malignancies like lymphoma and skin cancer. In the modern day, most patients with GN preferentially receive tacrolimus over cyclosporine.

### Genetic influences and therapeutic drug monitoring

Genetic influences affect the metabolism of tacrolimus, particularly certain variants of CYP3A5. Sequencing now permits the classification of individuals into “extensive”, “intermediate”, or “poor” metabolizers of tacrolimus, thus predicting the *in vivo* response to a given dose increase or decrease. Additionally, a clinical tool used to evaluate metabolizer status, in the absence of genetic testing, is the C0/D ratio, which is defined as the ratio of the trough concentration of tacrolimus in nanograms per milliliter (C0) and the daily dose of tacrolimus in milligrams (D). Patients with a lower C0/D ratio behave as extensive metabolizers, and such patients tend to have poorer graft outcomes and greater infectious complications after transplantation (22, 23). However, similar studies in GN are lacking.

Because of the narrow therapeutic window and interindividual differences in the metabolism of these drugs, therapeutic drug monitoring is essential to avoid toxicity. Tacrolimus is best monitored with a trough (C0) level, while cyclosporine is best assessed with a peak (C2) level (18). There are, however, limited studies on the ideal target level to be achieved in the treatment of various GN’s, and data are generally extrapolated from the transplant setting. Key Monitoring Checklist includes the following: BP, weight: Weekly initially, then monthly, Renal function: Serum creatinine, eGFR – 2 times/week initially, Electrolytes: K+, Mg2+, Ca2+ – Weekly, Blood glucose & HbA1c: Monthly or as clinically indicated, Liver function tests (LFTs): Monthly, Lipid profile: Every 3 months, Drug levels: Trough levels per protocol (depends on indication & time since transplant).

### Voclosporin - a novel calcineurin inhibitor:

Voclosporin is a second-generation CNI approved for use in lupus nephritis by the FDA in 2021 (21) based on results from the phase II AURA-LV and phase III AURORA trials (**Table 4**). For individuals whose baseline estimated glomerular filtration rate (eGFR) is at least 45 ml/min per 1.73 m², KDIGO recommends adding voclosporin to mycophenolate mofetil (MMF) and glucocorticoids for one year as initial lupus nephritis therapy. It is four times more potent than cyclosporine and does not require monitoring of drug levels to assess dose-response owing to a more consistent pharmacokinetic profile. Furthermore, there is less of a tendency to produce hypertension, dyslipidemia, and hyperglycemia compared to traditional CNI’s (24, 25). Notably, however, there have been no head-to-head comparisons between voclosporin and cyclosporine or tacrolimus (6) (**Table 5**).

**Table 4:** Indications for Voclosporin in Lupus Nephritis (KDIGO 2024) (7).

Clinical Setting	Indication	Remarks
Initial Therapy	Used with MPAA and glucocorticoids for active Class III/IV LN (± Class V)	- eGFR ≥45 ml/min/1.73 m²- Particularly in patients with nephrotic-range proteinuria
	Alternative for patients unfit for MPAA or cyclophosphamide	- Preferred in case of intolerance or fertility concerns
	May benefit patients with podocyte injury	- Especially if proteinuria is disproportionately high
Maintenance Therapy	Can be continued as part of triple immunosuppression regimen	- Supported by AURORA 1 & 2 trials
	Use in patients already treated with voclosporin in induction phase	- Maintain if initial response was favorable
Alternative Use	Consider if MPAA or azathioprine are contraindicated	- Option in limited access/resource settings

**Table 5:** Comparison of Calcineurin Inhibitors.

	Cyclosporine	Tacrolimus	Voclosporine
Chemical structure and origin	A cyclic polypeptide derived from *Tolypocladium inflatum*. One of the earliest CNIs introduced into clinical practice	A macrolide antibiotic derived from *Streptomyces tsukubaensis* Generally considered more potent than cyclosporine on a mg-per-mg basis.	A synthetic analog of cyclosporine with structural modifications designed to enhance potency and improve pharmacokinetics (PK)/pharmacodynamics (PD).
Mechanism of Action	binds cyclophilin to inhibit calcineurin	binds FK506-binding protein (FKBP) to inhibit calcineurin	bind cyclophilin to inhibit calcineurin
Pharmacokinetics and Pharmacodynamics	Requires therapeutic drug monitoring (TDM) to maintain effective and safe levels.	Requires TDM	Structural modifications increase binding affinity to the cyclophilin–calcineurin complex, resulting in a more consistent PK/PD relationship. Early data suggest that routine TDM may not be necessary for voclosporin (though clinical judgment and ongoing studies guide practice).
Adverse effects	Nephrotoxicity - increased fibrosis and tubular atrophy, hypertension, dyslipidemia, hyperglycemia, malignancy, hyperkalemia, hypomagnesemia, hyperglycemia gum hyperplasia, hirsutism, hyperlipidemia	Nephrotoxicity, increased fibrosis and tubular atrophy, hypertension, dyslipidemia, hyperglycemia, malignancy, hyperkalemia, hypomagnesemia, hyperglycemia, Neurotoxicity and alopecia	Similar to other CNIs – nephrotoxicity, hypertension
Use in Pregnancy	US FDA pregnancy category: C (animal studies have shown a risk to the fetus, but there are no adequate human studies. However, potential benefits may warrant use of the drug in pregnant women despite potential risks.	The current available data do not suggest an increased risk of major congenital malformations following *in utero* exposure to tacrolimus.	Contraindicated. This drug contains alcohol, which may cause harm to the fetus, such as central nervous system issues, behavioral problems, and impaired intellectual development.

FK506 refers to Tacrolimus. The designation “FK” in FK506 comes from the name of the company that first isolated the compound, Fujisawa Pharmaceutical Co., Ltd., which is now part of Astellas Pharma. The compound was discovered in 1984 from a soil sample collected in Japan. The number “506” is a sequential identifier used in the research process to label different compounds. Thus, “FK506” indicates it is the 506th compound studied by Fujisawa.

US FDA, US Food and Drug Administration.

## Anti-CD20 monoclonal antibodies

Rituximab, a type I chimeric anti-CD20 monoclonal antibody, is now recommended therapy for ANCA vasculitis and membranous nephropathy, and has roles in the management of steroid-dependent nephrotic syndrome and lupus nephritis. Recently, obinutuzumab, a type II anti-CD20 monoclonal antibody, has had positive trial results in lupus nephritis. The NOBILITY trial demonstrated that adding Obinutuzumab to standard therapies could significantly improve kidney function outcomes in patients with proliferative lupus nephritis (26–28) (**Tables 6**, **7**).

**Table 6:** Difference between Rituximab and Obinutuzumab.

*Drug *	*Rituximab*	*Obinutuzumab*
*Type of monoclonal antibody*	Chimeric	Humanized
*Mechanism of action*	activates AKT, BAD, and NOTCH1.	induces apoptosis through SYK phosphorylation
*B-cell depletion*	Depletes B-cells	Depletes B-cells more effectively than rituximab
*Antibody-dependent cell-mediated cytotoxicity*	Enhances this process	Enhances this process more compared to rituximab
*Clinical use*	Steroid resistant nephrotic syndrome, Membranous Nephropathy, Lupus nephritis	Used to treat patients resistant to Rituximab
*Anti-drug antibody*	More likely to be affected by anti-drug antibodies	Less likely to be affected by anti-drug antibodies

AKT: also known as Protein Kinase B (PKB). The name AKT is derived from “Ak mouse strain thymoma.” BAD: Bcl-2-associated death promoter. It is a pro-apoptotic member of the Bcl-2 protein family. NOTCH1: Notch homolog 1, translocation-associated (Drosophila). NOTCH1 is a member of the NOTCH family of proteins, which are involved in cell differentiation processes.

**Table 7:** Indications for Rituximab in Lupus Nephritis (KDIGO 2024) (7).

Clinical Setting	Indication	Remarks
Initial Therapy	For refractory lupus nephritis	Rituximab may be considered in patients with active Class III or IV lupus nephritis who have an inadequate response to initial standard-of-care therapy.
Adjunct Therapy	Add-on to standard therapy	Rituximab can be used in combination with mycophenolate mofetil or cyclophosphamide for patients with persistent disease activity despite standard treatment.

### Adverse effects: infusion-related reactions

Given the chimeric nature of the rituximab, infusion-related reactions have commonly been described, and may manifest as an innocuous skin rash or as life-threatening anaphylaxis. It is therefore common protocol to pre-medicate patients receiving rituximab with dexamethasone and anti-histamines, to avoid these reactions.

### Infectious complications

Depletion of B-cells may result in flares of hepatitis B, hepatitis C and tuberculosis. Thus, all patients who are planned for rituximab should be screened for these infections and appropriately treated before administration of rituximab. Notably, screening for hepatitis B should include detection of HBsAg-negative, anti-HBc-positive patients, as antiviral prophylaxis is recommended for these patients when B-cell depleting therapy is administered (29).

Hypogammaglobulinemia has also been reported, especially in patients with ANCA-associated vasculitis, and can sometimes lead to recurrent infections (30). Some protocols recommend measuring baseline immunoglobulin levels prior to rituximab therapy, and re-assessment every 6-12 months for the duration of rituximab therapy. However, even very low immunoglobulin levels sometimes do not correlate with clinical infectious episodes, and therefore clear thresholds for therapeutic administration of intravenous immunoglobulin have not yet been defined (31).

Other rare infectious complications that have been described with rituximab are late-onset neutropenia and an increased predilection to progressive multifocal leukoencephalopathy (32).

### Rituximab-induced serum sickness

It is thought that B-cell lysis after administering rituximab results in the release of various intracellular antigens that then result in antigen-antibody complexes, producing a type III hypersensitivity reaction. It typically presents 1-2 weeks after rituximab exposure and manifests as a serum-sickness syndrome, with fever, purpura and arthritis. It is managed with corticosteroids and anti-histamines but could recur if the drug is re-challenged (32).

### Reduced-dose rituximab

There has been some interest in studying the utility of reduced-dose rituximab in the treatment of various GN’s. It has been proposed that the cumulative therapeutic dose can be safely reduced without affecting the clinical efficacy of the drug through CD19-targeted dosing (33, 34). This would likely reduce several of the infectious complications of rituximab by reducing exposure to the drug.

## Belimumab

Belimumab is a recombinant human IgG-1λ monoclonal antibody against B lymphocyte stimulator (BLyS), also termed as B-cell activating factor (BAFF). Despite already being in wide use for extra-renal systemic lupus erythematosus, it has only recently been included by KDIGO as an add-on to standard-of-care for the treatment of lupus nephritis, based on the results of the BLISS-LN trial (6).

It is administered as monthly intravenous injections for up to 2.5 years, and may therefore be preferable for patients with prior non-compliance to therapy. The BLISS-LN trial did not demonstrate any safety signals of concern, as compared to placebo, and infection rates were similar in both groups (35).

Based on the KDIGO 2024 Clinical Practice Guideline for the Management of Lupus Nephritis, the indications for belimumab in the treatment of lupus nephritis (LN) are as follows (7):

1. Initial therapy for active Class III or Class IV LN (with or without a membranous component):

- Belimumab in combination with:

   o Mycophenolic acid analogs (MPAA), or
   o Low-dose intravenous cyclophosphamide

- Should be given along with glucocorticoids
- This combination is a Level 1B recommendation

(Strong recommendation; moderate certainty of evidence)

2. Preferred option in special situations:

- Triple immunosuppressive regimen (Belimumab + Glucocorticoids + MPAA or reduced-dose cyclophosphamide) may be preferred in:

   o Patients with repeated kidney flares
   o Patients at high risk for progression to kidney failure    due to severe CKD

3. Maintenance therapy:

- After achieving remission, belimumab may be continued as part of triple immunosuppressive maintenance therapy (with MPAA or azathioprine and low-dose steroids).

## Drugs affecting the complement cascade

### Monoclonal antibodies against complement C5

Eculizumab and ravulizumab are monoclonal antibodies against complement factor C5, thereby preventing formation of the membrane attack complex, which is the final effector of complement-mediated injury. These drugs are used extensively in complement-mediated thrombotic microangiopathy.

The major complication of C5 inhibition is the expected increased susceptibility to infection, especially those caused by encapsulated organisms such as Neisseria. Therefore, all patients receiving eculizumab therapy should be vaccinated for meningococcus. However there is a variable immunological response after complement blockade, and antibiotic prophylaxis is recommended for the duration of therapy (36).

### Avacopan

Avacopan is a C5a receptor inhibitor, and has been used as a steroid sparing agent in the treatment of ANCA-associated vasculitis. In the ADVOCATE trial avacopan, as compared with prednisolone, resulted in fewer infections overall (68.1% vs 75.6%), fewer serious infections (13.3% vs 15.2%) and fewer serious opportunistic infections (3.6% vs 6.7%). Interestingly, there were no cases of Neisseria meningitidis, because unlike C5 inhibition, avacopan does not impede the formation of the membrane attack complex, and therefore immune defenses against encapsulated organisms remain preserved (37).

### Iptacopan

This is an oral inhibitor of factor B, which therefore attenuates the activity of the alternative complement pathway. It has been studied in IgA nephropathy, and the recent APPLAUSE-IgAN phase 3 has resulted positive interim results in terms of proteinuria reduction at 9 months of therapy. It was noted that there was no increased risk of infection compared to placebo, and infections with encapsulated bacteria occurred in less than 0.5% of patients (38).

Iptacopan, marketed as Fabhalta, has received the following U.S. Food and Drug Administration (FDA) approvals:

- December 2023: Approved for treating adults with paroxysmal nocturnal hemoglobinuria (PNH) ​ (39, 40).
- August 2024: Granted accelerated approval for reducing proteinuria in adults with primary immunoglobulin A nephropathy (IgAN) at risk of rapid disease progression ​ (41).
- March 2025: Approved as the first treatment for adults with complement 3 glomerulopathy (C3G) (42).

## Financial burden

The financial burden of immunosuppressive and immunomodulatory drugs used in the treatment of glomerular diseases can be significant, affecting both individual patients and healthcare systems. Many of these medications, such as calcineurin inhibitors, mycophenolate mofetil, and biologics like rituximab, are not only expensive but may also require continuous use over extended periods to manage conditions like lupus nephritis and other forms of glomerulonephritis effectively. The costs are further compounded by the need for regular monitoring and management of potential side effects, which can necessitate additional medication or treatment interventions. For instance, the management of side effects such as bone marrow suppression, infections, or nephrotoxicity can lead to increased healthcare utilization, including hospital admissions and the need for expensive supportive care. Furthermore, the high costs of these drugs can limit access in lower-income regions where glomerulonephritis prevalence is notably high, thereby exacerbating disparities in health outcomes. This financial impact extends beyond the direct costs of the drugs to include the broader economic effects of managing chronic kidney disease, such as lost productivity and other long-term social costs (43).

In conclusion, the treatment of glomerular diseases with immunosuppressive therapies such as glucocorticoids, mycophenolate mofetil (MMF), and cyclophosphamide presents a complex clinical challenge (**Table 8**). While these medications can significantly suppress disease activity and improve patient outcomes, they also carry potential risks, including gastrointestinal symptoms, bone marrow suppression, and increased susceptibility to infections. Regular monitoring and careful dose adjustment are critical to minimize these risks and manage side effects effectively. Additionally, advancements in pharmacological approaches, like the use of enteric-coated mycophenolate sodium and tailored dosing regimens, offer new avenues to enhance tolerability and maintain therapeutic efficacy. As the global burden of glomerular diseases grows, ongoing research and development of safer, more effective treatments are essential to address the evolving needs of this patient population, ultimately aiming to improve their quality of life and clinical outcomes.

**Table 8:** Summary of drugs, mechanism of action and adverse effects.

Drug	Mechanism of Action	Common Side Effects
Glucocorticoids	Anti-inflammatory and immunosuppressive properties	Increased risk of infection, bone disorders, dysglycemia, obesity, hypertension, psychosis, gastrointestinal bleeding, cataracts, cardiovascular risks
Mycophenolate Mofetil (MMF)	Inhibits inosine monophosphate dehydrogenase affecting purine synthesis of B and T lymphocytes	Viral infections (e.g., herpes, cytomegalovirus), bone marrow suppression, leukopenia, gastrointestinal upset, headaches, fatigue, eczema, hair loss
Cyclophosphamide	Alkylating agent that causes DNA cross-linking and breaks, leading to apoptosis of lymphocytes	Bone marrow and gonadal damage, increased risk of cancers, bladder inflammation and scarring, infertility in males, ovarian failure in females
Calcineurin Inhibitors (Cyclosporine, Tacrolimus, Voclosporin)	Inhibit calcineurin leading to T-cell activation suppression	Nephrotoxicity, increased fibrosis and tubular atrophy, hypertension, dyslipidemia, hyperglycemia, neurotoxicity (tacrolimus), hypertrichosis and gingival hyperplasia (cyclosporine) infections, hyperkalemia, hypomagnesemia, malignancy
Rituximab, Obinituzumab	Chimeric anti-CD20 monoclonal antibody leading to B-cell depletion	Infusion-related reactions, flares of viral infections, hypogammaglobulinemia, late-onset neutropenia, serum sickness
Azathioprine	Metabolizes to 6-mercaptopurine, forming 6-thioguanine nucleotides. These nucleotides disrupt nucleic acid synthesis and cell replication, primarily reducing T-lymphocyte proliferation.	Bone marrow suppression, gastrointestinal side effects, hepatotoxicity, increased susceptibility to infections, hypersensitivity reactions, pancreatitis, increased risk of malignancy –particularly, lymphoma and skin cancer
Eculizumab and Ravulizumab	Monoclonal antibody against C5	increased susceptibility to infection from capsulated organisms
Avacopan	C5a receptor inhibitor	Gastrointestinal issues, hepatotoxicity, headaches, hypertension, upper respiratory infections, and hypersensitivity.
Iptacopan	Factor B inhibitor	Headache, gastrointestinal disturbances, infections, fatigue, or dizziness. Long term safety is not yet established
Belimumab	Fully human monoclonal antibody, that works by targeting and inhibiting the action of the soluble form of the B-lymphocyte stimulator (BLyS), also known as BAFF (B-cell activating factor).	Infusion reaction, infections, hypersensitivity reactions, gastrointestinal symptoms, psychiatric symptoms
